# Mechanosensitive Molecular Networks Involved in Transducing Resistance Exercise-Signals into Muscle Protein Accretion

**DOI:** 10.3389/fphys.2016.00547

**Published:** 2016-11-17

**Authors:** Emil Rindom, Kristian Vissing

**Affiliations:** ^1^Section of Sport Science, Department of Public Health, Aarhus UniversityAarhus, Denmark; ^2^Department of Biomedicine, Aarhus UniversityAarhus, Denmark

**Keywords:** mechanotransduction, PLD-PA, BMP-Smad, Rho-STARS, Rheb

## Abstract

Loss of skeletal muscle myofibrillar protein with disease and/or inactivity can severely deteriorate muscle strength and function. Strategies to counteract wasting of muscle myofibrillar protein are therefore desirable and invite for considerations on the potential superiority of specific modes of resistance exercise and/or the adequacy of low load resistance exercise regimens as well as underlying mechanisms. In this regard, delineation of the potentially mechanosensitive molecular mechanisms underlying muscle protein synthesis (MPS), may contribute to an understanding on how differentiated resistance exercise can transduce a mechanical signal into stimulation of muscle accretion. Recent findings suggest specific upstream exercise-induced mechano-sensitive myocellular signaling pathways to converge on mammalian target of rapamycin complex 1 (mTORC1), to influence MPS. This may e.g. implicate mechanical activation of signaling through a diacylglycerol kinase (DGKζ)-phosphatidic acid (PA) axis or implicate integrin deformation to signal through a Focal adhesion kinase (FAK)-Tuberous Sclerosis Complex 2 (TSC2)-Ras homolog enriched in brain (Rheb) axis. Moreover, since initiation of translation is reliant on mRNA, it is also relevant to consider potentially mechanosensitive signaling pathways involved in muscle myofibrillar gene transcription and whether some of these pathways converge with those affecting mTORC1 activation for MPS. In this regard, recent findings suggest how mechanical stress may implicate integrin deformation and/or actin dynamics to signal through a Ras homolog gene family member A protein (RhoA)-striated muscle activator of Rho signaling (STARS) axis or implicate deformation of Notch to affect Bone Morphogenetic Protein (BMP) signaling through a small mother of decapentaplegic (Smad) axis.

## Introduction: muscle myofibrillar protein accretion

Skeletal muscle contractile properties can be negatively affected with prolonged inactivity and/or muscle wasting disease, leading to severe deteriorations in muscle myofibrillar mass, muscle strength, and mobility (Clark, 2009). Consequently, knowledge on resistance exercise-induced mechanisms involved in stimulating a positive net muscle myofibrillar turnover is important. This turnover is dictated by a balance between protein synthesis and protein degradation, but is contended to be primarily driven by regulation of muscle protein synthesis (MPS) (Atherton and Smith, 2012) and with resistance exercise-induced MPS accentuated by amino acid supplementation (Rasmussen and Phillips, 2003; Wolfe, 2006).

Intriguingly, mechanical force changes inherent of resistance exercise are assumed to exert regulatory action on mechanisms involved in MPS. With regards to resistance exercise, this immediately advance questions such as; (a) how mechanical force changes inherent of resistance exercise is sensed; (b) how resistance exercise-induced mechano-sensing molecules can affect biochemical signaling to directly activate MPS and; (c) how resistance exercise-induced mechano-sensing can exert influence on MPS by means of regulating net supply of muscle myofibrillar gene transcripts to the ribosomal machinery.

Below, we will first summarize the burden of proof that advocate for the significance of mechanotransducing mechanisms. We will then summarize some emerging knowledge on how force changes with resistance exercise may be sensed by mechanosensitive molecules to activate biochemical signaling for MPS and/or muscle myofibrillar gene transcription. Most of the current knowledge on mechanotransduction has been retrieved through employment of *in vitro* or animal models, whereas less information exists from human resistance exercise studies. Consequently, most of the findings presented, originate from non-human studies. However, human studies that provide support for the implication of similar mechanisms in human skeletal muscle are included, with attention on the influence of resistance exercise modality and intensity.

## Growth factor-independent mTORC1 activation for MPS in adult skeletal muscle suggests an important role of mechanotransducing mechanisms

Mechanistic target of rapamycin complex 1 (mTORC1) is regarded as a nodal point for integration of various stimulators, such as growth factors, nutrients and mechanical forces, to activate downstream signaling for muscle protein translation initiation (Laplante and Sabatini, 2012). Its importance has been justified in different model systems (Bodine et al., 2001; Hornberger et al., 2004; Sandri, 2008; Goodman et al., 2011; Goodman, 2014) and activation of mTORC1 signaling in human skeletal muscle has been shown to be associated with increased MPS during post-exercise recovery from traditional high-intensity resistance exercise (Dreyer et al., 2006, 2008; Drummond et al., 2008) as well as fatiguing low-intensity blood-flow restricted resistance exercise (Fujita et al., 2007; Fry et al., 2010).

mTORC1-inhibitor, rapamycin, has been employed in cell culture and rodent models, to assess whether mTORC1 can be considered outright necessary for activation of MPS subsequent to force changes, with studies on acute responses to single-treatment intervention immediately supporting this (Bodine et al., 2001; Fingar et al., 2002, 2004; Hornberger et al., 2004; Kubica et al., 2005), while results from studies on basal MPS are less conclusive (Kubica et al., 2005; Drummond et al., 2009). The few human studies that have utilized rapamycin in investigation of resistance exercise-induced mTORC1 signaling and MPS provide support that rapamycin exert similar effect upon high-intensity resistance exercise (Drummond et al., 2009) and low intensity blood-flow restricted resistance exercise (Gundermann et al., 2014).

Downstream from mTORC1, 70 kDa ribosomal S6 kinase (P70S6K) seem to provide a stronger proxy of signaling for resistance exercise-induced MPS, than mTORC1 (Baar and Esser, 1999; Nader and Esser, 2001). It can therefore be speculated that P70S6K may be activated by mechanical stress in an mTORC1-independent manner (Klossner et al., 2009), but as the previous studies on this have not included mTORC1 loss-off-function analysis, this requires further investigation.

It has been previously contended that resistance exercise drives muscle hypertrophy by promoting an increase in systemic growth factors like IGF-1, which via its receptor activate a PI3K-Akt-mTORC1 signaling axis to enhance MPS (Yan et al., 1993; Coleman et al., 1995; Goldspink et al., 1995; Adams and Haddad, 1996; Musarò et al., 2001; Rommel et al., 2001). However, more recent studies contradict that systemic growth hormones are vital for driving resistance exercise-induced MPS in the adult muscle. This opposite contention is based on findings including (a); the demonstration of loading-induced PI3K-independent mTORC1 activation through utilization of genetic loss of functions models or PI3K-inhibitor Wortmannin (Hornberger et al., 2004, 2007; O'Neil et al., 2009; Miyazaki et al., 2011), (b); the demonstration that mechanical overload in rodents with a dominant-negative IGF-I receptor do not abolish hypertrophy (Spangenburg et al., 2008), (c); the demonstration that resistance exercise regimens utilizing high vs. low resistance exercise training volumes can accentuate increases in systemic plasma growth factors, but without simultaneously accentuating mTORC1 activation, MPS and/or hypertrophy (West et al., 2009, 2010), and, (d); the observation that human resistance exercise in the fasting state does not seem to activate Akt, yet still promote activation of mTORC1 (Deldicque et al., 2008; Vissing et al., 2013a). The recent skepticism toward an important role of exercise-induced systemic hormones has led to the proposal that intrinsic mechano-sensitive molecules constitute more pivotal drivers of MPS.

## How is a change in force during resistance exercise sensed by the muscle cell?

With regards to mechano-sensing, several excellent reviews has previously addressed how mechanical force imposed on a muscle cell can be envisaged to induce conformational changes on specific muscle proteins interlinking extracellular matrix (ECM) (e.g., collagen or laminin), sarcolemmal (e.g., integrins or cadherins), focal adhesion (e.g., FAK or actin filaments), costamere (e.g., dystrophin and vinculin) and/or Z-disk (e.g., titin or phospholipase D) proteins (Patel and Lieber, 1997; Burkholder, 2007; Ingber, 2010).

For the sake of simplicity, one may distinguish between tensile and/or compressive stresses that are expectedly generated during resistance exercise/muscle loading. In accordance, if a muscle cell is exposed to tensile stress in one direction, it creates a state of internal stress that causes constriction in the perpendicular plane, while compressive stress in the transverse plane will develop internal stresses that cause lengthening and therefore, once again, a tensile stress (Burkholder, 2007). In essence, tensile stress therefore likely constitute one very important trigger of deformation of mechano-sensing muscle proteins during resistance exercise, with different modes of resistance exercise, likely imposing partially different effects on specific mechano-sensing proteins. Thus, with regards to contraction mode, tensile stress inherent of eccentric contractions may e.g., be sensed by sarcolemmal/transmembranal receptor proteins (such as integrins) that tie the extracellular matrix (ECM) to focal adhesion complex proteins (Chicurel et al., 1998; Ingber, 2010; Olson and Nordheim, 2010), whereas concentric contractions can be speculated to produce a tensile stress that is predominantly sensed by proteins inherent of the sarcomere (Hornberger et al., 2006b). As for fatiguing low intensity resistance exercise with or without blood flow restriction, this has been shown to produce accumulation of metabolites and/or increased muscle water retention (Qin and Hu, 2014; Farup et al., 2015). Accordingly, with reference to the literature on endothelial cells, shear stress inherent of such fluid shifts may be sensed by some of the same ECM and focal adhesion proteins that are speculated to sense a mechanical stress on muscle cells during resistance exercise (Hirakawa et al., 2004; Petzold et al., 2009). However, evaluation on whether eccentric vs. concentric resistance exercise affect mechano-sensing molecules differentially is challenging, as it inevitable necessitate simultaneous considerations on the intensity with which the specific contraction modes are performed (i.e., the higher the relative intensity/load inherent of a given resistance exercise regime, the higher the expected stimulatory effect on mechanosensitive proteins; Eliasson et al., 2006).

## How can a mechanical signal elicited by resistance exercise be transduced to biochemical signaling for muscle protein synthesis?

**Figure 1:**
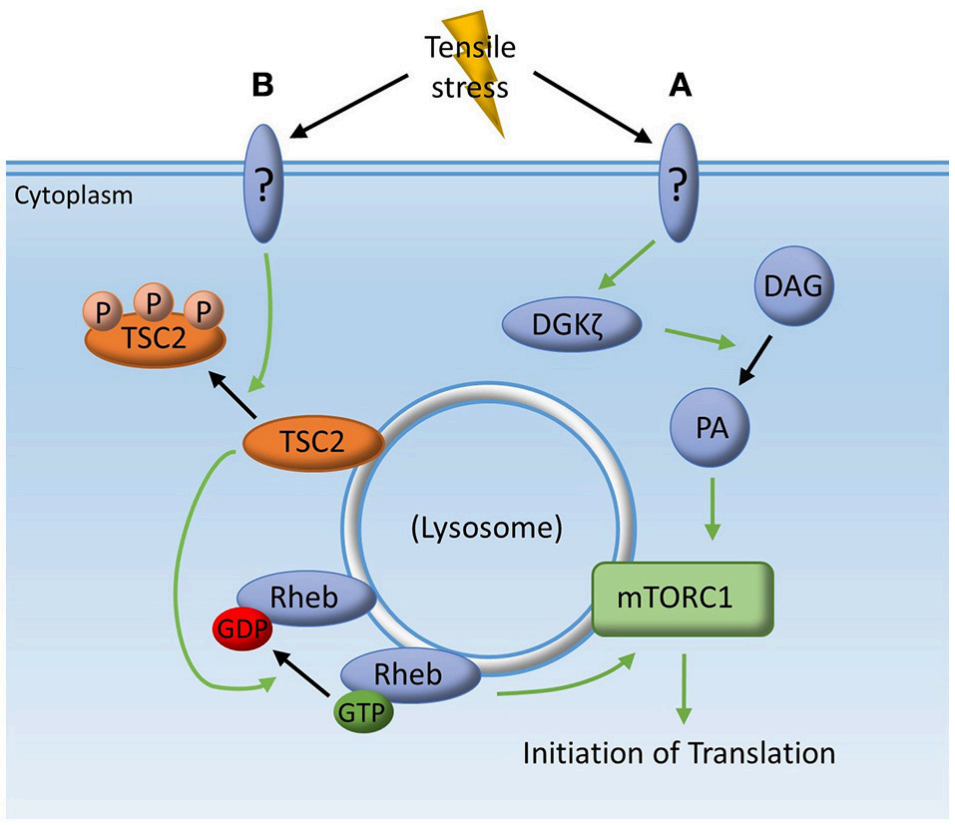
**Mechanotransduction for muscle protein synthesis**. Tensile stress inherent of mechanical deformation may stimulate muscle protein synthesis through; **(A)** yet unidentified mechanosensing proteins acting on the zeta isoform of diacylglycerol kinase (DGKζ), resulting in the conversion of diacylglycerol (DAG) to phosphatidic acid (PA) which then directly activates the mechanistic target of rapamycin complex 1 (mTORC1); **(B)** an unidentified kinase phosphorylating the tuberous sclerosis complex-2 (TSC2) which is then translocated away from the lysosome allowing Ras homolog enriched in brain (Rheb) to be in its guanosine triphosphate (GTP) bound state which can then directly activate mTORC1.

Knowledge on resistance exercise-induced mechanotransduction for MPS in humans is still relatively sparse. Some indicatory information can e.g., be retrieved from comparative studies on the differential effects of differentiated exercise (MacNeil et al., 2010; Vissing and Schjerling, 2014; Petriz et al., 2016). However, the knowledge on specific mechanotransducing mechanisms presented below (see also Figure 1), is predominantly based on findings from *in vitro* or animal studies.

### Phosphatidic acid (PA)

Phosphatidic Acid is a diacyl-glycerophospholipid that is primarily synthesized from phosphatidylcholine (PC) by the enzyme phospholipase D (PLD). In the unstimulated state, α-actinin (in the z-band of the sarcomere) associates with and inhibits phospholipase D (PLD) (Park et al., 2000; Hornberger et al., 2006a). It is speculated that phospholipase D dissociates from α-actinin during mechanical deformation (Hornberger et al., 2006a). Such dissociation relieve the inhibition of phospholipase D, leading to its hydrolysis and the formation of PA. Phosphatidic Acid has been demonstrated to directly activate mTORC1 signaling (Fang et al., 2001; Park et al., 2002) by binding to the FKBP12-rapamycin binding (FRB) domain of mTOR (Fang et al., 2001; Veverka et al., 2008; You et al., 2012). In accordance, exogenous PA (Foster, 2007; O'Neil et al., 2009; You et al., 2012) as well as overexpression of the PA-generating enzymes PLD1 (Jaafar et al., 2013), LPAATθ (Tang et al., 2006) and DGKζ (Avila-Flores et al., 2005; You et al., 2014) have been demonstrated to activate mTORC1 signaling, suggesting an important role for PA in the regulation of cellular growth. More recently, the role of PLD1 in controlling the mechanically induced changes in PA and mTORC1 signaling has been evaluated using the specific PLD-inhibitor 5-fluoro-2-indolyl des-chlorohalopemide (FIPI) (You et al., 2014). As FIPI was not able to prevent increases in PA or mTORC1 signaling in rodent muscles following passive stretch, this suggests a limited role of PLD1. However, other experiments using overexpression of the zeta isoform of diacylglycerol kinase (DGKζ) has been demonstrated to produce hypertrophy, which was largely inhibited by rapamycin, while knockdown of the DGKζ-gene almost completely abolished the passive stretch-induced increase in PA and impaired activation of mTORC1 signaling (You et al., 2014). These results collectively suggest that DGKζ may link a tensile stress inherent of resistance exercise to increases in PA and mTORC1 signaling.

With regards to human studies, one resistance exercise training study has employed oral supplementation of PA, producing an increase in lean body mass compared to placebo (Hoffman et al., 2012).

### Rheb

Ras homolog enriched in brain (Rheb) is a GTP-binding protein that expresses GTPase activity (Aspuria and Tamanoi, 2004). When in its GTP-bound state, Rheb has been reported to directly activate mTOR signaling (Sancak et al., 2007; Sato et al., 2009). Both Rheb and its downstream target mTOR have been reported to be highly enriched in the lysosome. Furthermore, evidence is emerging that controlling mTOR-association to the lysosome is an important step in regulating mTOR activity (Sengupta et al., 2010; Zhao et al., 2012). In accordance, it has been suggested that Rheb is regulated by the tuberous sclerosis complex-2 (TSC2) also found at the lysosome. In basal conditions, TSC2 stimulates Rheb's GTPase activity, which, in turn, leads to conversion of active GTP-Rheb into inactive GDP-Rheb (Huang and Manning, 2008), thereby repressing mTORC1 activity. A recent study have shown TSC2 phosphorylation and translocation from the lysosome as well as activation of lysosome-associated mTORC1 following electrically stimulated lengthening contractions in mice (Jacobs et al., 2013). The TSC2 phosphorylation occurred on different sites than the Thr1462 site previously described to be important for Akt-dependent phosphorylation (Inoki et al., 2002). This suggests that a yet unidentified mechano-sensing protein is responsible for TSC2 phosphorylation and mTORC1 activation following tensile stress inherent of the lengthening contractions (Jacobs et al., 2013). In this regard, a recent study on the effects of inhibition of integrin-associated Focal adhesion kinase (FAK), suggests that FAK can affect TSC2 phosphorylation and a subsequent Rheb mediated activation of mTORC1, leading to concomitant activation of P70S6K and MPS (Crossland et al., 2013). Interestingly, the integrin-FAK axis also seems to be involved in the regulation of myofibrillar gene expression (i.e., Rho-STARS pathway—see below; Zhao et al., 2007).

## How can a mechanical signal elicited by resistance exercise influence transcriptional events inherent of muscle protein accretion?

**Figure 2:**
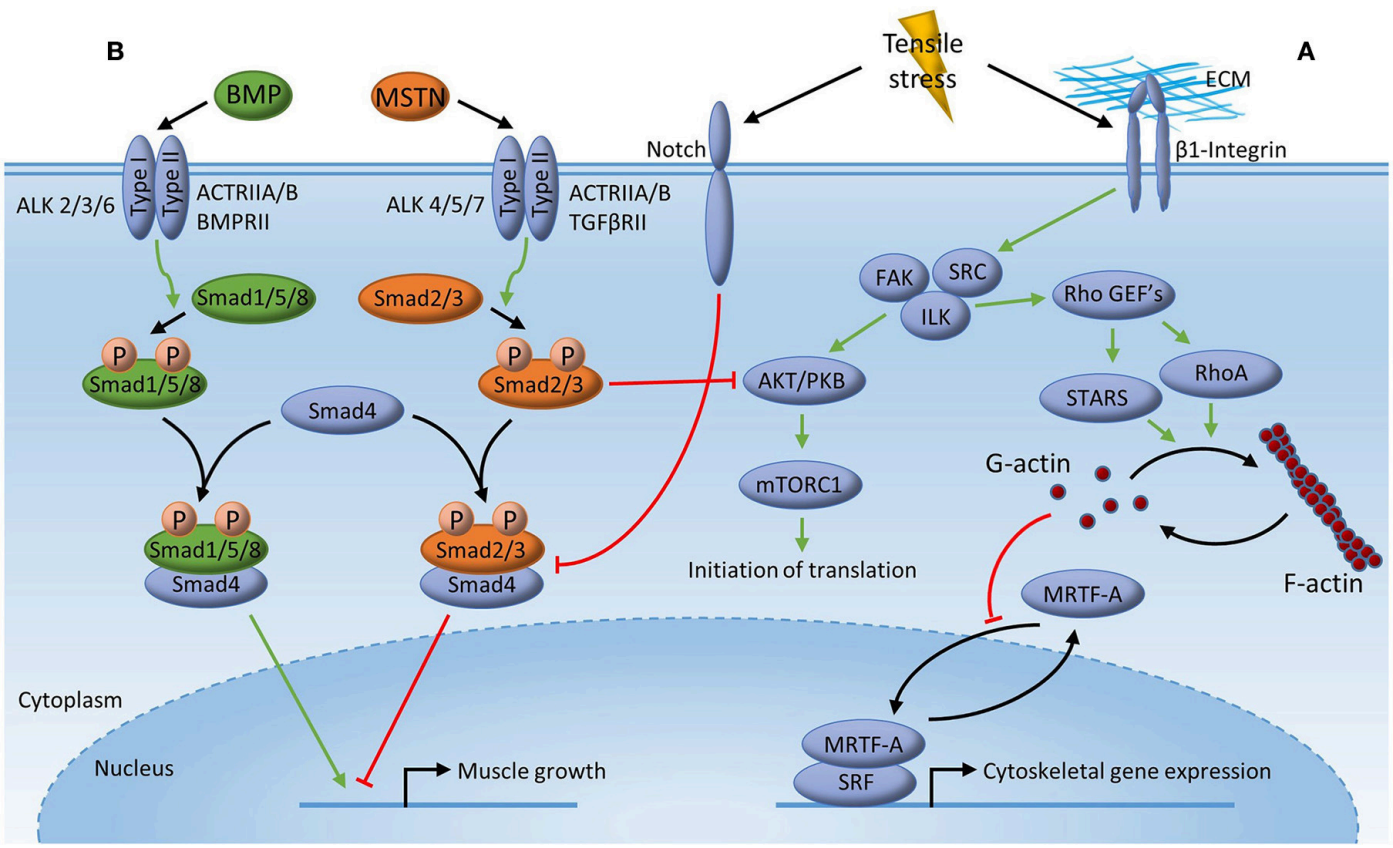
**Mechanotransduction for muscle mRNA transcription**. Tensile stress inherent of mechanical deformation may stimulate muscle mRNA transcription through; **(A)** deformation of membrane-associated β1-Integrin activating focal adhesion kinase (FAK), integrin-linked kinase (ILK) and SRC, which then promotes activation of striated muscle activator of Rho signaling (STARS) and Ras homolog gene family member A (RhoA) through Rho guanine nucleotide exchange factors (GEFs) leading to polymerization of globular actin (G-actin) into filamentous actin (F-actin). Release of cytoplasmic G-actin from myocardin-related transcription factor (MRTF) then allows MRTF to translocate to the nucleus to act as a co-transcription factor with transcription factor serum response factor (SRF), leading to gene expression of multiple muscle myofibrillar and cytoskeletal genes; **(B)** competitive inhibition of Myostatin (MSTN) signaling by Bone Morphogenetic Protein (BMP) signaling through the common mediator small mother of decapentaplegic 4 (Smad4). Binding of MSTN to its receptor, leads to phosphorylation of Smad2/3 enabling formation of a transcriptional complex with Smad4, which then translocate to the nucleus to modulate transcriptional events resulting in impaired muscle growth. BMP leads to phosphorylation of Smad1/5/8 resulting in the possible formation of a Smad1/5/8-Smad4 transcriptional complex resulting in expression of genes important for muscle growth. Tensile stress inherent of mechanical deformation limits smad2/3 signaling through the membrane-associated protein Notch thereby allowing Smad1/5/8 signaling resulting in muscle accretion.

While direct regulation of MPS obviously constitutes an important rate-limiting level of myocellular muscle protein accretion, MPS also depends on the magnitude and/or rate of delivery of muscle myofibrillar mRNA to the ribosomes. In this regard, recent knowledge on specific pathways is presented below that may link mechanical stress to myofibrillar gene expression (see also Figure 2).

### Rho-STARS

Actin dynamics constitute an element of muscle contraction, with actin exhibiting an interchange between monomeric/globular (G-actin) and polymeric/filamentous (F-actin) forms (Chen et al., 2000). This mechanism is proposed to influence signaling via the transcription factor, serum response factor (SRF), which in turn possess transcriptional control of a multitude of muscle genes adhering to proliferation and differentiation events and/or cell growth (Olson and Nordheim, 2010; Braun and Gautel, 2011). More specifically, in unstimulated cells, cytosolic G-actin is bound to the SRF transcriptional co-activator myocardin-related transcription factor-A (MRTF-A), thereby preventing interaction of MRTF-A with SRF. However, the release of G-actin from MRTF-A upon G-actin polymerization into F-actin following mechanical stimulation, results in the nuclear translocation of MRTF-A allowing it to associate with SRF to enhance SRF transcriptional activity (Kuwahara et al., 2005, 2007; Visegrády and Machesky, 2010).

Further upstream from MRTF, control is suggested to be mediated by the Ras homolog gene family member A protein (RhoA) and the striated muscle activator of Rho signaling (STARS, also known as actin-binding Rho-activating protein, ABRA). Both proteins can activate actin-associated proteins following mechanical signals, with activation of STARS or RhoA leading to G-actin polymerization and SRF-mediated gene transcription (Arai et al., 2002; Liu et al., 2003). Studies using STARS knockdown/suppression (Arai et al., 2002; Kuwahara et al., 2005; Wallace and Russell, 2013) or overexpression (Wallace and Russell, 2013) has produced decreases and increases in SRF transcriptional activity and SFR-associated mRNA levels, respectively. Furthermore, studies utilizing inhibition of RhoA has been demonstrated to reduce SRF transcriptional activity following STARS activation (Arai et al., 2002), suggesting that STARS activation of transcription is partly mediated by a RhoA-dependent mechanism.

Interestingly, RhoA is activated by β1 Integrin upon mechanical stress (McClung et al., 2004), a process also involving activation of Rho guanine exchange factors by integrin-linked kinases such as ILK, FAK, and SRC (Huveneers and Danen, 2009), with ILK and FAK seemingly also able to activate Akt (Xia et al., 2004; Wang et al., 2008).

*In vivo*, force changes have been shown to activate the actin-MRTF-SRF pathway and to promote increases in RhoA protein expression in overloaded rat muscle (McClung et al., 2003; Sakuma et al., 2003). In humans, single-bout high intensity resistance exercise have been observed to produce increased STARS gene expression (Lamon et al., 2009, 2013; MacNeil et al., 2010) and prolonged resistance training have been observed to produce increased gene expression and/or nuclear protein levels of several members of the Rho-STARS-SRF pathway (Lamon et al., 2009; Vissing et al., 2013b). As judged from analysis of gene expression, eccentric resistance exercise modality may constitute a stronger driver of these responses (Lamon et al., 2009, 2013; MacNeil et al., 2010). However, as increased STARS protein expression has been observed exclusively with concentric resistance, further investigation on this is required (Vissing et al., 2013b).

### BMP-smads

The transforming growth factor-beta (TGFβ) family of ligands, such as Myostatin/TGFβ, has been shown to negatively affect the regulation of muscle mass due to receptor-mediated activation of a class of effector molecules known as small mother of decapentaplegic (Smad) proteins (Lee and McPherron, 2001; Lee et al., 2005). This may be counteracted by cytokines referred to as Bone Morphogenetic Proteins (BMPs) also acting on Smad proteins in a manner influenced by potentially the mechano-sensing transmembranal protein, Notch (MacKenzie et al., 2013). More specifically, binding of the Myostatin or TGFβ ligand to the activin type II receptors (ActRIIA and ActRIIB) and TGFβ receptors (TGFβRII), triggers the recruitment and activation of the tyrosine kinases activin type I receptors (ALK4, ALK5, and ALK7), resulting in phosphorylation of specific Smad proteins (Smad2 and Smad3, Smad2/3), which enables the Smad proteins to form a transcriptional complex with the Smad4 protein. The Smad2/3-Smad4 complex then translocate to the nucleus where it modulates chromatin structure in a manner to decrease expression of genes associated with muscle growth (Gaarenstroom and Hill, 2014). Interestingly, it has more recently been discovered, that BMPs can activate a Smad1/5/8 complex that can influence activity of Smad2/3 (Sartori et al., 2013; Winbanks et al., 2013). Accordingly, Smad 1/5/8 phosphorylation occur in consequence of binding of BMP to BMP- (BMPRII) or activin type II receptors (ActRIIA and ActRIIB) leading to subsequent recruitment and activation of other activin type I receptors than myostatin/TGFβ (namely ALK2, ALK3, and ALK6; Walsh et al., 2010). Phosphorylation of the BMP-dependent Smad proteins has been proposed to enable the formation of a transcriptional complex with Smad4 in a similar manner to what is observed for Smad2/3. Yet, whereas the Smad2/3-Smad4 complex negatively influences muscle growth-related genes, activation of the Smad1/5/8-Smad4 complex produce increased expression of genes associated with cell growth and differentiation (Miyazono and Miyazawa, 2002). Evidence that a balance between muscle atrophy and hypertrophy depends on the recruitment of the shared mediator Smad4 to either the Myostatin vs. the BMP signaling pathways, is based on observations from transgenic mouse models. In accordance, inhibition of Smad1/5/8 (by overexpression of a BMP-inhibitor, Noggin) increased denervation-mediated muscle atrophy compared to wildtype. This inhibition of BMP signaling was accompanied by increased recruitment of phosphorylated Smad2/3 binding to Smad4 and translocation to the nucleus (Sartori et al., 2013). Moreover, activation of BMP signaling through utilization of adeno-associated viral vector (AVV)-mediated overexpression of a constitutively active type I BMP receptor (caALK3) was observed to prevent muscle atrophy as well as to stimulate hypertrophy in denervated muscle fibers (Winbanks et al., 2013). Furthermore, rapamycin was shown to counteract muscle accretion promoted by BMP overexpression (Winbanks et al., 2013).

Interestingly, transmembranal protein Notch has been shown to increase in response to high frequency electrical stimulation and has been suggested to possess a negative regulatory effect on TGFβ signaling by inhibiting Smad 2/3, thereby allowing Smad 1/5/8 signaling for hypertrophy (MacKenzie et al., 2013).

A present, little is known on how resistance exercise may favor activation of the Smad1/5/8-Smad4 complex, but transcriptome analysis from our own previous analysis serve to support that certain BMP and Smad isoforms are transcribed in adult human skeletal muscle and that certain isoforms exhibit differential expression with differentiated exercise (Vissing and Schjerling, 2014). Thus, human resistance exercise can be speculated to produce BMP to function in an autocrine manner.

## Challenges to address in elucidation of mechanotransducing mechanisms with differentiated resistance exercise

Most of the knowledge on potential mechanotransduction mechanisms summarized in the current review is derived from *in vitro* or animal models. The advantages of such models are that they offer genetic homogeneity, manipulation through genetic engineering and easy use of compounds such as wortmanin and rapamycin. On the other hand, the stimulation protocols utilized in those studies typically do not ideally mimic resistance exercise regimens as they are genuinely practiced in humans. In this regard, animal models that can render voluntary resistance exercise possible would offer an experimental advantage. Moreover, animal models that employ surgical ablation models and electrical stimulation likely impose non-exercise related stressors and/or omit true stressors inherent of voluntary human resistance exercise. In this regard, non-intervention control models would improve the ability to deduce potential separate effects of invasive procedures and/or dietary premises inherent of stimulation protocols that potentially obscure interpretation of results. Human studies offer volitional resistance exercise, but imply genetic heterogeneity and a similar need to for non-intervention control models to control for e.g., systemic effectors. Furthermore, loss of function and/or overexpression manipulation is difficult to apply to human studies. Consequently, results from human studies are often most quite descriptive unless of comprehensive comparative designs. However, comparative studies introduce difficult considerations on sample size and whether/how to match exercise protocols for volume, intensity and/or whether exercise is performed to a state of fatigue. In this regard, increased use of administration of rapamycin and wortmanin in future human resistance exercise studies might help provide more causal information.

As previously suggested, the ability to address these challenges would benefit from a multi-site consortium of scientists, to facilitate translational studies through use of non-human and human model systems (Neufer et al., 2015).

## Author contributions

All authors listed, have made substantial, direct and intellectual contribution to the work, and approved it for publication.

### Conflict of interest statement

The authors declare that the research was conducted in the absence of any commercial or financial relationships that could be construed as a potential conflict of interest.
